# Political views and organizational distrust affect rural residents’ willingness to share personal data for COVID-19 contact tracing: A cross-sectional survey study

**DOI:** 10.1017/cts.2023.33

**Published:** 2023-03-27

**Authors:** Jennifer B. McCormick, Margaret Hopkins, Erik B. Lehman, Michael J. Green

**Affiliations:** 1 Department of Humanities, Penn State College of Medicine, Hershey, PA, USA; 2 Department of Public Health Sciences, Penn State College of Medicine, Hershey, PA, USA; 3 Department of Medicine, Penn State College of Medicine, Hershey, PA, USA

**Keywords:** Data sharing, contact tracing, survey, COVID-19, empirical research

## Abstract

**Background:**

We aimed to examine the attitudes of Pennsylvania rural residents toward data sharing in the setting of the COVID-19 pandemic. Specifically, we were interested in better understanding their willingness to provide personal information for contact tracing to public health staff investigating COVID-19 cases, as well as their concerns. We used a validated scale to describe the influence of distrust of healthcare organizations on their attitudes.

**Methods:**

We mailed 4000 surveys to rural residents identified from the electronic medical record of a healthcare system in central Pennsylvania. Data were entered into a REDCap database and analyzed using descriptive summaries, and both binomial and multivariable logistic regression.

**Results:**

Binomial logistic regression showed that both distrust in healthcare organizations and political values influence respondents’ willingness to share information with contact tracers as well as their concerns about sharing personal data. When our multivariable model was applied, political values remained and were consistently associated with willingness to share and concerns about sharing their data.

**Conclusion:**

This study is a first step in eliciting rural residents’ willingness to share personal data for contact tracing by public health officials. Understanding and addressing rural residents’ willingness to share personal data and their concerns about sharing those data will help public health officials identify effective strategies for managing COVID-19 and future pandemics in rural communities. By involving community members at the ground level, public health staff can ensure residents’ buy-in for the need to collect their personal data, thereby helping to mitigate the public health crises.

## Introduction

In December 2019, the World Health Organization (WHO) was informed of several cases of pneumonia of unknown cause in Wuhan City, China. By early January 2020, it was determined that the cause was a newly discovered coronavirus, soon to be known as COVID-19. While the first case in the USA was detected in an individual in Washington State in January 2020, there soon were community outbreaks across the country, and in early March 2020, the US government declared a public health emergency due to the outbreak. Many states declared states of emergency and implemented contact tracing by public health staff to let individuals know that they may have been exposed to someone with confirmed or probable COVID-19.

Public health departments have long used contact tracing as a means of tracking, limiting, and decreasing the spread of infectious disease. However, the rapid transmission of COVID-19 and high rates of infection from asymptomatic individuals have made manual contact tracing infeasible, and contact tracing through mobile technologies was promoted as a faster, less labor-intensive, and more efficient means of controlling viral spread [1,2]. University research groups, governments, and technology companies responded by building mobile applications that leveraged and deployed cell phone data [3–5]. By December 2020, almost four dozen countries had introduced or proposed contact tracing apps and encouraged people to download and use them [5]. While some countries accessed people’s cell phone data without their permission, no US governments at the state or federal level used contract tracing apps in a systematic way [6].

Unsurprisingly, given current levels of distrust toward authority, Americans display ambivalence toward digital contact tracing apps. In one survey, two-thirds of respondents indicated that they would download and install a coronavirus tracking app, while another poll found that only 43% of respondents with cell phones would use an infection-tracking app [7,8]. That percentage went up to between 70 and 80% in another study, but only if the app had some guarantee of privacy protection or accuracy [9]. However, in two studies comparing various surveillance policies, respondents’ support for digital contact tracing only ranged between 40% and 49% and never was the top choice [2,8]. Researchers also found that support for and likely downloading of tracing apps varied depending on who developed or provided them – with more support for apps developed by public health agencies such as the Centers for Disease Control and Prevention (CDC) as opposed to those from technology companies and startups [5,7]. Given estimates that containment requires at least 60% of a population using the apps, these conflicting attitudes call into question whether digital contact tracing is a viable mechanism for controlling the spread of COVID-19 [1,2].

Few studies have investigated the attitudes of rural residents about governmental efforts to collect data – whether from digital sources or provided verbally to contact tracers – for tracking the spread of a highly infectious disease. Having an understanding of those attitudes is critical for public health departments, given that rural areas pose unique challenges for managing a pandemic. For instance, on average, Americans living in rural areas are older, sicker, and more likely to die from heart disease and cancer than urban residents [10,11]. Rural residents’ higher rates of chronic illnesses including obesity, Chronic Obstructive Pulmonary Disorder (COPD), and diabetes likewise increase their vulnerability to diseases even absent a fast-moving pathogen like COVID-19 [12,13]. While rural geography would seem to create a natural and protective social distancing for residents, such isolation also typically means a limited and inadequate healthcare infrastructure [14]. That can translate to: fewer local testing facilities; hospitals with limited resources such as ventilators and dedicated Intensive Care Units (ICUs); and “hospital deserts” where residents have to travel up to 50 miles to reach a hospital [12,13,15]. Furthermore, rural residents are more likely to be uninsured or underinsured than their urban counterparts [15].

Likewise, little is known about rural residents’ attitudes toward public health organizations and institutions such as the CDC and state departments of health. Broadly, Americans have been shown to have mixed support for these entities. According to a report on 12 national polls on the public health system, Americans were generally dissatisfied with state public health departments but satisfied with the CDC [16]. A more recent study comparing responses to public opinion polls conducted in 2018 and 2020 showed increased support for and trust in public health departments, likely the result of their role in the COVID-19 pandemic [17]. This support, however, appears to be mercurial. According to a May 2021 poll, only slightly more than half of Americans have a great deal of trust in the CDC and only one in three Americans have a great deal of trust in the National Institutes of Health (NIH) and Food and Drug Administration [18]. None of these polls and studies specifically examined the views of rural residents. Knowing whether rural residents trust public health officials and the healthcare organizations working with them will provide critical insight into whether people will comply with governmental recommendations for the control of infection and disease [19]. Compliance depends on trust in and support of government health recommendations and policies as demonstrated by studies involving past public health crises [20].

The quantitative study results reported here are a first step in understanding rural residents’ willingness to share personal data and concerns about sharing those data in the setting of the COVID-19 pandemic. First, it examines rural participants’ willingness to share personal data in two instances involving hypothetical public health measures – that is, providing cell phone location data as well as providing data on recent face-to-face contacts to public health staff investigating COVID-19 cases. This study also examines the associations between (1) rural residents’ trust in and distrust of healthcare organizations and their willingness to provide data to public health staff, and (2) rural residents’ sociodemographic characteristics and their willingness to provide personal data.

Specifically, we address the following research questions:

- How do respondents’ sociodemographic characteristics influence their willingness to provide personal data to public health staff investigating COVID-19?
- How do participants’ sociodemographic characteristics influence their concerns about personal data being shared with public health staff investigating COVID-19?
- How do participants’ trust in and distrust of healthcare organizations influence their willingness to provide personal data to public health staff investigating COVID-19?
- How do participants’ trust in and distrust of healthcare organizations influence their concerns about sharing personal data with public health staff investigating COVID-19?

## Methods

### Ethics Statement

The Institutional Review Board of the Pennsylvania State University approved the study. Since this was a mail survey study, respondents’ consent was implied when they returned the completed survey. All participants were over 18 years of age.

### Study Participants

We used the electronic medical records of an academic healthcare institution in central Pennsylvania to obtain names and addresses of patients who had visited an outpatient clinic or been an inpatient within the prior 3 years, were 18 years of age or older, and who resided in a community defined as “rural.” Central Pennsylvania was selected for convenience; the authors are members of the university to which the healthcare institution is affiliated. We chose 3 years as our cutoff for inclusion because we wanted to include in our sample patients who had relatively recently received care at the healthcare institution. This was important since several of the survey questions (not presented here) were specific to the healthcare institution. For this study, rural was defined as a community: (1) with a population density less than the statewide density of 284 persons per square mile, or (2) where the total population is less than 2500, unless more than 50% of the population lives in an urbanized area as defined by the US Census Bureau [21]. Eighteen of the 28 counties in the healthcare institution’s catchment area have been designated Appalachian. In general, rural Appalachian counties have lower median household incomes, higher rates of poverty, and lower levels of education and employment than rural counties outside Appalachia [22]. These demographics were similar to many of our counties.

Seventeen of the 18 counties designated in Appalachia and in the institution’s catchment area have lower median household incomes than Pennsylvania’s median household income [21]; 10 have higher poverty rates than Pennsylvania’s [22]; and 15 have higher unemployment rates than Pennsylvania’s [22]. Of relevance to this study, in 16 of the counties, registered Republican voters in 2020 outnumbered registered Democratic voters, sometimes by as much as 3-to-1 [21].

A letter, summary explanation of research, incentive (a $2 bill), and survey were mailed to 4000 patients randomly selected from the 6000+ patients matching our criteria. No electronic mechanism (e.g., email, patient portal) was used to distribute the survey. Recruitment occurred in mid-October 2020. The survey was open through early January 2021 and available in English only.

### Survey Development

The survey included four statements about contact tracing and three about intent to receive the COVID-19 vaccine. These statements were developed based on news reports about people’s concerns that providing personal data for contact tracing could erode privacy protections and result in government and businesses’ increased access to those data [23,24]. Response options were “Strongly agree,” “Agree,” “Neither agree nor disagree,” “Disagree,” and “Strongly disagree.” Participants were advised they could skip any questions. The survey also had a section involving demographics, two validated scales measuring trust and distrust, and two other sections with questions related to general data sharing in the context of research. The results from the general data sharing questions are not presented here.

For bivariable and multivariable analysis, the outcome variables of interest were the four statements about contact tracing: “I would be willing to share my cell phone location data with public health officials investigating COVID-19 cases”; “I am concerned that without my permission, my cell phone location data could be shared with public health staff investigating COVID-19 cases”; “I would be willing to share data (names, addresses, and phone numbers) of people with whom I have had recent in-person contact with public health staff investigating COVID-19 cases”; “I am concerned that the personal data I share with public health staff may be used for purposes other than investigating COVID-19 cases.”

The independent variables included sociodemographic factors (i.e., age, gender, race/ethnicity, educational level, employment status, and household income) and questions asking participants to self-characterize their health status, religiosity, and political values. Two validated scales measuring trust were included: the 9-item Distrust in Healthcare Organizations scale (DHO) and the 22-item World Assumptions Questionnaire (WAQ) (see Supplemental Information for scoring) [25–27]. The DHO measures people’s perceptions of healthcare organizations’ values, such as honesty and motives, and issues of competence from a broad prespective – that is, not individual hospitals or clinics but all the organizations that are part of healthcare including county and state public health departments. The DHO has been used to investigate whether patients’ distrust of healthcare organizations influences their use of healthcare resources and observance of healthcare recommendations [28,29]. Questions include: “Healthcare organizations put making money above patients’ needs”; “Healthcare organizations provide excellent medical care”; “Healthcare organizations do their best to make patients’ health better”; and “Healthcare organizations make too many mistakes.” Because county and state public health departments can serve as communities’ healthcare organizations, people’s perceptions of those organizations can serve as a proxy for their attitudes toward complying with public health investigations and recommendations.

We used the WAQ to approximate how trusting an individual is. This enabled us to ensure that the DHO was measuring distrust in healthcare organizations and not simply distrust in general.

The WAQ includes questions to measure respondents’ perceived controllability and predictability of life events. Questions include: “I don't feel in control of the events that happen to me”; “It is ultimately up to me to determine how events in my life will happen”; “For the most part, I believe people are good”; and “It is difficult for me to take most of what people say at ‘face value.’” It also measures whether respondents trust other people and are able to see goodness in them [26,27].

### Data Analysis

All study variables were summarized prior to analysis to determine their distributions. Because of small cell counts, some categories within certain predictor variables including age, religiosity, education, health status, and employment were combined (Supplemental Information). Likert scales were considered not continuous for the purposes of analysis. Ordinal logistic regression was initially attempted on the Likert scale outcome variables based on level of agreement in relation to sharing of personal data and cell phone location, but given numerous violations with the proportional odds assumption, the outcome variables were ultimately collapsed to binary variables with categories of “agree” or “disagree,” where “agree” included “agree” and “strongly agree,” and “disagree” included “neither agree nor disagree,” “disagree,” and “strongly disagree.” We chose to put “neither agree nor disagree” into the negative category of “disagree” as we focused on the positive (“agree”) as our outcome. We did not include “race/ethnicity” as a variable as our sample was predominantly White (95%).

Bivariate binomial logistic regression was then applied to all independent variables with all outcome variables to determine the unadjusted effect of each independent variable on each outcome variable. A multivariable model including all of the independent variables was then fit for each outcome variable. Prior to multivariable modeling, the independent variables were tested for multicollinearity using variance inflation factor (VIF) statistics. Any predictor variables with VIF statistics > 5 would be eliminated from the model, but all independent variables were retained. Odds ratios and 95% confidence limits were used as the effect size to quantify the magnitude and direction of any significant associations between the independent variables and outcome variables. The fit of the multivariable models was assessed using the Hosmer–Lemeshow goodness-of-fit test. The *p*-values of the same predictor variable analyzed against the four outcome variables were adjusted for multiple testing (four tests) using the false discovery rate method in both bivariate and the multivariable analyses. All analyses were performed using SAS version 9.4 (SAS Institute, Cary, NC).

## Results

The response rate was 19.5%. Most of the participants self-identified as White with a small percentage identifying as Hispanic. (See Table 1.) Nearly two-thirds of respondents were older than 60 years and almost half were retired. Religion or spirituality was extremely important or very important to more than 60% of respondents. While fewer than 10% self-identified as very liberal/liberal politically, more than 40% of respondents self-identified as very conservative/conservative politically. More than 40% of respondents also had a high school education or less. Of those who chose to provide income data, about one-third had an annual household income of $50,000 or less (see Table 1).

**Table 1.** Characteristics of respondents (N = 758) for survey study conducted in fall 2020 of rural patients in central Pennsylvania

Characteristic	Frequency	Percent
Race		
White	719	94.85
Black or African American	2	0.26
American Indian or Alaska Native	2	0.26
Asian	1	0.13
Mixed	3	0.40
Other	5	0.66
Prefer not to answer	10	1.32
Missing	16	2.11
Ethnicity		
Hispanic	8	1.06
Non-Hispanic	706	93.14
Prefer not to answer	16	2.11
Missing	28	3.69
Gender		
Male	315	41.56
Female	423	55.80
Non-binary	1	0.13
Prefer not to answer	3	0.40
Missing	16	2.11
Health		
Poor/fair	190	25.07
Good	307	40.50
Very good/excellent	234	30.87
Missing	27	3.56
Age		
<40	94	12.40
41–60	193	25.46
>60	452	59.63
Prefer not to answer	4	0.53
Missing	15	1.98
Importance of religion, spirituality		
Extremely/very	450	59.37
Moderately	156	20.58
Slightly/not at all	117	15.44
Prefer not to answer	13	1.72
Missing	22	2.90
Education		
High school/GED or less	302	39.84
Technical school or some college	176	23.22
College graduate	144	19.00
Graduate or professional school	107	14.12
Missing	29	3.83
Employment status		
Not employed	116	15.30
Employed	265	34.96
Retired	348	45.91
Missing	29	3.83
Total household income		
$30,000 or less	121	15.96
$31,000–$50,000	123	16.23
$51,000–$70,000	124	16.36
$71,000–$100,000	95	12.53
Greater than $100,000	91	12.01
Prefer not to answer	174	22.96
Missing	30	3.96
Political values		
Very conservative, conservative	320	42.22
Moderate	166	21.90
Liberal, very liberal	56	7.39
Other	15	1.98
Prefer not to answer	175	23.09
Missing	26	3.43
DHO score (9–45, higher=less trust)	691	24.12 ± 5.40
Missing	67	8.84
WAQ score (0–66, higher=more trustworthy)	621	30.44 ± 8.00
Missing	137	18.07

DHO, Distrust in Healthcare Organizations; WAQ, World Assumptions Questionnaire.

*Note*: DHO and WAQ scores use Mean ± Standard Deivation.

### DHO and WAQ

The scores from the DHO scale and the WAQ were not significantly correlated and were treated as continuous variables in our analysis. The overall mean score for the DHO was 24.12 (SD 5.40). Higher scores reflect lower levels of trust (score range is 9–45, maximum score is 45). We used tertiles to delineate among low (bottom tertile), medium (middle), and high distrust (top). Respondents with high distrust (*n* = 248; 35.9%) outnumbered those with medium distrust (*n* = 228; 32.9%) and those with low distrust (*n* = 215; 31.1%). The mean scores for DHO tertiles were Top = 29.67 ± 3.61, Middle = 23.56 ± 1.09, and Bottom = 18.33 ± 2.85.

The overall mean for the WAQ was 30.44 (SD 6.33). Higher scores on the WAQ reflect higher levels of trust (score range is 0–66, maximum score is 66). Here, we also used tertiles to describe low (bottom tertile), medium (middle), and high (top) trust. Respondents with medium level of trust (*n* = 238; 38.3%) outnumbered those respondents with low trust (*n* = 190; 30.6%) and those with high trust (*n* = 193; 31.6%). The mean scores for the WAQ tertiles were Top = 37.43 ± 3.53, Middle = 30.45 ± 1.74, and Bottom = 23.33 ± 3.80.

### Frequency Data

Table 2 presents frequency data on participants’ responses to the four outcome questions on contact tracing. Only about one-fourth of respondents agreed/strongly agreed with the statement, “I would be willing to share my cell phone location data with public health staff investigating COVID-19 cases.” That percentage increased to 44% when asked if they would be willing to share data of people with whom they had recent in-person contact. A similar percentage (44%) of respondents had concerned that their data would be used for purposes other than investigating COVID-19 cases. Slightly more respondents (51%) worried their data would be accessed without their permission (see Table 2 and Table S1 in Supplemental Information).

**Table 2.** Frequency and percentages of outcome variables for survey study conducted in fall 2020 of rural patients in central Pennsylvania

Question	Frequency (%)
I would be willing to share my cell phone location data with public health staff investigating COVID-19 cases	
Yes	209 (27.57%)
No	513 (67.68%)
Missing	36 (4.75%)
I am concerned that without my permission, my cell phone location data could be shared with public health staff investigating COVID-19 cases.	
Yes	383 (50.53%)
No	339 (44.72%)
Missing	36 (4.75%)
I would be willing to share data (names, addresses, phone numbers) of people with whom I have had recent in-person contact with public health staff investigating COVID-19 cases.	
Yes	333 (43.93%)
No	394 (51.98)
Missing	31 (4.09%)
I am concerned that the personal data I share with public health staff may be used for purposes other than investigating COVID-19 cases.	
Yes	394 (51.98%)
No	337 (44.46%)
Missing	27 (3.56%)

### Bivariable Analysis

Respondents who were trusting by nature as measured by the WAQ were more likely to agree to share cell phone data and provide others’ contact information. This is in contrast to individuals whose DHO scores were high and who, therefore, are more likely to not trust healthcare organizations and public health entities. These respondents were more likely to be unwilling both to share their cell phone location data and to provide names and contract information of people with whom recent in-person contact had occurred (see Table S2 in Supplemental information).

Respondents who self-identified as being either conservative or moderate politically also were less likely to support providing cell phone data to public health staff as were those for whom religion is extremely/very important. Lower educational levels (high school graduation or less) also was associated with unwillingness to provide information to public health investigators.

High DHO scores also was associated with respondents’ concerns that their cell phone data would be used without their permission and that their personal data would be used for purposes other than investigating COVID-19. This was true as well for respondents who self-described as conservative politically and very religious. However, respondents whose nature is more trusting as measured by high WAQ scores were less likely to fear their cell phone data would be accessed without their permission and had fewer concerns their data would be used for purposes other than COVID-19 tracking.

### Multivariable Analysis

A multivariable model was created using demographics, DHO score, and WAQ score as predictor variables. We did not use race/ethnicity since our sample was primarily White, non-Hispanic. In the text, we include only those predictor variables that have a significant influence (*p*-value ≤ to 0.05) on the four outcome questions (see Table 3).

**Table 3.** Characteristics of respondents in relation to contact tracing and data sharing as adjusted odds ratios (95% confidence intervals), multivariate analysis, for survey study conducted in fall 2020 of rural patients in central Pennsylvania, corrected for multiple analyses

Characteristic	I would be willing to share my cell phone location data with public health staff investigating COVID-19 cases.	I am concerned that without my permission, my cell phone location data could be shared with public health staff investigating COVID-19 cases.	I would be willing to share data (names, addresses, phone #s) of people with whom I have had recent in-person contact with public health staff investigating COVID-19 cases.	I am concerned that the personal data I share with public health staff may be used for purposes other than investigating COVID-19.
Gender				
Male	1	1	1	1
Female	1.02 (0.66–1.57), *p* = 0.933	0.79 (0.53–1.16), *p* = 0.446	1.03 (0.74–1.65), *p* = 0.839	0.58 (0.39–0.85), ***p* = 0.023**
Health				
Poor/fair	1	1	1	1
Good	1.46 (0.85–2.52), *p* = 0.680	0.79 (0.49–1.29), *p* = 0.696	0.92 (0.56–1.53), *p* = 0.755	0.81 (0.50–1.32), *p* = 0.527
Very good/excellent	0.62 (0.33–1.16), *p* = 0.279	1.06 (0.62–1.82), *p* = 0.835	0.65 (0.37–1.15), *p* = 0.279	0.90 (0.53–1.55), *p* = 0.835
Age				
<40	1	1	1	1
41–60	0.89 (0.44–1.82), *p* = 0.750	1.67 (0.90–3.08), *p* = 0.409	0.87 (0.46–1.65), *p* = 0.750	1.21 (0.66–2.23), *p* = 0.750
>60	2.38 (1.08–5.25), *p* = 0.078	1.17 (0.58–2.36), *p* = 0.872	2.15 (1.04–4.44), *p* = 0.078	1.00 (0.51–1/99), *p* = 0.993
Importance of religion, spirituality				
Extremely/very	0.48 (0.26–0.86), ***p* = 0.058**	1.45 (0.83–2.51), *p* = 0.379	0.85 (0.49–1.50), *p* = 0.577	1.30 (0.76–2.21), *p* = 0.456
Moderately	0.63 (0.33–1.21), *p* = 0.221	2.03 (1.09–3.73), *p* = 0.101	0.59 (0.31–1.10), *p* = 0.194	1.22 (0.67–2.22), *p* = 0.526
Slightly/not at all	1	1	1	1
Education				
High school/GED or less	0.60 (0.30–1.20), *p* = 0.198	0.57 (0.30–1.06), *p* = 0.154	0.51 (0.27–0.95), *p* = 0.137	1.27 (0.70–2.31), *p* = 0.440
Technical school or some college	1.06 (0.54–2.11), *p* = 0.858	0.56 (0.30–1.07), *p* = 0.321	0.70 (0.36–1.34), *p* = 0.557	1.17 (0.63–2.19), *p* = 0.813
College graduate	1.19 (0.60–2.33), *p* = 0.806	0.58 (0.31–1.09), *p* = 0.372	0.81 (0.43–1.56), *p* = 0.806	0.93 (0.50–1.71), *p* = 0.806
Graduate or professional school	1	1	1	1
Employment status				
Not employed	1	1	1	1
Employed	0.53 (0.28–1.04), *p* = 0.254	1.40 (0.78–2.54), *p* = 0.525	1.09 (0.59–2.01), *p* = 0.778	1.25 (0.70–2.23), *p* = 0.598
Retired	0.54 (0.26–1.12), *p* = 0.397	1.29 (0.67–2.51), *p* = 0.461	1.50 (0.77–2.94), *p* = 0.461	1.28 (0.67–2.44), *p* = 0.461
Total household income				
$30,000 or less	1.21 (0.51–2.88), *p* = 0.804	1.63 (0.74–3.60), *p* = 0.804	0.83 (0.37–1.84), *p* = 0.804	0.91 (0.42–1.95), *p* = 0.804
$31,000–$50,000	1.14 (0.52–2.51), *p* = 0.988	0.99 (0.49–2.03), *p* = 0.988	0.67 (0.32–1.40), *p* = 0.988	1.12 (0.56–2.25), *p* = 0.988
$51,000–$70,000	0.90 (0.41–1.99), *p* = 0.802	1.48 (0.73–2.99), *p* = 0.547	0.84 (0.41–1.71), *p* = 0.802	1.72 (0.86–3.42), *p* = 0.496
$71,000–$100,000	1.98 (0.94–4.18), *p* = 0.296	1.02 (0.51–2.02), *p* = 0.962	1.09 (0.54–2.22), *p* = 0.962	0.84 (0.43–1.65), *p* = 0.962
Greater than $100,000	1	1	1	1
Prefer not to answer	0.60 (0.26–1.40), *p* = 0.325	1.55 (0.74–3.25), *p* = 0.325	0.40 (0.19–0.87), *p* = 0.080	1.40 (0.68–2.89), *p* = 0.359
Political values				
Very conservative, conservative	0.20 (0.09–0.44**), *p* < 0.001**	3.92 (1.77–8.69), ***p* = 0.011**	0.15 (0.06–0.36), ***p* < 0.001**	2.75 (1.30–5.78), ***p* = 0.008**
Moderate	0.45 (0.21–0.97), *p* = 0.084	1.87 (0.84–4.15), *p* = 0.168	0.36 (0.15–0.88), *p* = 0.084	1.39 (0.66–2.93), *p* = 0.394
Liberal, very liberal	1	1	1	1
Prefer not to answer	0.19 (0.08–0.43), ***p* < 0.001**	4.75 (2.07–10.90), ***p* < 0.001**	0.16 (0.06–0.40), ***p* < 0.001**	2.84 (1.30–6.23), ***p* = 0.009**
DHO score	0.77 (0.62–0.95), ***p* = 0.036**	1.15 (0.95–1.39), *p* = 0.188	0.92 (0.76–1.11), *p* = 0.383	1.25 (1.04–1.51), *p* ** = 0.036**
WAQ	1.10 (0.92–1.33), *p* = 0.400	0.85 (0.72–1.00), *p* = 0.187	1.09 (0.93–1.29), *p* = 0.400	0.94 (0.80–1.11), *p* = 0.471

DHO, Distrust in Healthcare Organizations; WAQ, World Assumptions Questionnaire.

*Odds ratios, 95% confidence limits, and p-values from multivariable binomial logistic regression adjusted for all variables in the model (and table).

Strongly Disagree/ Disagree/ Neither; 0 = Agree/Strongly Agree.

Bold text denotes statistically significant.

In multivariable analysis, respondents’ DHO scores and conservative political orientation continued to be associated with unwillingness to share cell phone data with public health investigators. Respondents’ distrust of healthcare organizations, however, was not a factor in whether they were willing to share the contact information for others. Rather those who self-identified as conservative politically were more likely to be unwilling to share information about their recent in-person contacts. (See Table 3.)

Accessing cell phone data without asking permission seemed to be a concern for respondents who identified as politically conservative. However, respondents with high DHO scores were more likely to be concerned their personal data would be used for purposes other than investigating COVID-19 cases. This was also true for those who self-identified as politically conservative or who chose not to indicate political orientation (see Table 3).

## Conclusion

In our survey of rural patients living in the catchment area of an academic medical center located in Pennsylvania, the majority of respondents had considerable reservations about sharing their cell phone location data with public health staff investigating COVID-19 cases. Our respondents also had concerns that their cell phone data would be accessed and used without their permission. More than half of our participants worried that their personal data would be used for purposes other than tracking COVID-19 cases. Paradoxically, when asked to share the names, addresses, and phone numbers of people with whom they had recent in-person contact, almost half expressed willingness to do so. While unwilling to share their own personal data, those respondents appeared more willing to share others’ data.

Across bivariable and multivariable analyses, only one factor was consistently predictive of how an individual would respond to the contact tracing questions: self-identified political values. Our survey, conducted during the Trump presidential administration, indicates that those who identified as conservative or very conservative were less willing to share cell phone data, less likely to share information about their recent in-person contacts, and more likely to be concerned their data would be shared without their permission and used for purposes other than contact tracing.

This is not surprising given that political partisanship has been found to be a factor in many Americans’ beliefs about the severity of COVID-19; necessity for and compliance with mitigating measures such as mask wearing; and willingness to be vaccinated [29,30,33]. Numerous studies have found that self-declared conservatives and Republicans generally do not comply with recommendations and mandates for two behaviors – namely, staying-at-home and socially distancing to reduce public exposure to and transmission of the virus [30–33]. According to one study, the influence of sociodemographics such as age and geography pales in comparison with political ideology for adherence to social distancing guidelines [32]. Partisanship also has been found to account for refusal to change travel plans, avoid large gatherings, and self-quarantine [34]. Our findings suggest another health behavior subject to partisanship – namely, willingness to provide personal data for contact tracing. Unlike respondents who self-described as liberal, those who self-described as very conservative or conservative were less likely to willingly engage in contact tracing.

Political partisanship in the context of health-related behaviors predates the COVID-19 pandemic [35]. Participants’ political values proved significant in a 2005 study investigating public attitudes toward mandatory health behaviors in the event of biological warfare involving smallpox [20]. However, in contrast to our findings, Democrats and Independents in that study were more leery than their Republican counterparts of government health policies, perceiving they would threaten individual liberties. As the authors note, it was unclear whether this occurred because Republicans tend to be more supportive than Democrats of government antiterrorism policies or because at that time Republicans held political power [20]. Indeed, studies have noted that Democrats had fewer concerns than Republicans about Ebola during the Obama administration [32,34].

Political partisanship has also been found to extend to trust in or distrust of science in general, including public health officials and organizations. In a study examining trust in science between 1974 and 2010, while those self-identifying as conservative started out with a higher level of trust than liberals and moderates, they ended the time period with the lowest level of trust [36]. Those self-identifying as conservative also have been found to have the lowest level of trust in the scientific community and to regard scientists and scientific institutions with suspicion [37]. More recently, in a study using cell phone data to determine compliance with stay-at-home orders, findings demonstrated that residents of Republican and conservative-leaning counties were less likely to comply with the stay-at-home policy than residents of Democratic-leaning counties. The authors argue that the lack of compliance reflects people’s distrust of science and public health policies for crisis mitigation [30].

That said, some of the distrust of biomedical and public health sciences that has arisen during the COVID-19 pandemic might reflect the initial uncertainties about the severity of the virus and the effectiveness of mitigation measures. For some Americans, changes in policies and recommendations were not understood as responses to more knowledge but as evidence that COVID-19 was a hoax, death rates exaggerated, and public health authorities not to be trusted and their recommendations not to be followed. In our study, participants’ distrust of public health officials investigating COVID-19 cases was suggested by the association of the DHO with two of our outcome variables. Not only were participants unwilling to allow public health officials to access their cell phone location data, but respondents also were concerned their data would be used for purposes other than controlling viral spread.

While political orientation proved a significant predictor in multivariable analysis, distrust of healthcare organizations was significant in the bivariable analysis. Researchers have found that rural residents generally are less trusting of healthcare systems for a variety of reasons including skepticism of outsiders and expert high turnover of providers and concerns about discrimination and stigma when accessing healthcare [38–41]. That said, the relationship between distrust in healthcare organizations and contact tracing that we identified may also be related to rural residents’ concerns about protection of personal privacy. One of Americans’ most often cited concerns about digital contact tracing centers on privacy of personal data and potential erosion of security protecting those data [2,9]. Additionally, people worry that their digital data will not be deleted when the pandemic ends and will then be used for purposes such as government surveillance, a concern shared by over half of our respondents [3,5].

In the initial weeks of the COVID-19 pandemic, urban counties generally experienced higher rates of infections and mortality than rural counties and urban residents generally adopted recommended health behaviors to control viral spread [40,42–44]. It has been suggested that in those first months, rural residents may have perceived COVID-19 as an urban plight with their rurality reducing the risk of infection and consequently, the need to comply with public health-oriented behavior change [11,42]. In subsequent surges, rural populations have accounted for higher rates of per capita morbidity, hospitalization, and mortality as well as greater cumulative case and death rates [11,45]. Even so, large segments of rural populations – particularly those who are White, self-identify as conservative politically, and do not trust medical and health experts – generally have not adopted public health-recommended prevention behaviors such as mask wearing and avoiding public spaces and crowds [40,42,43,46]. Our findings suggest that another prevention behavior – namely, sharing of personal information with public health officials for contact tracing – also is not endorsed by rural residents.

Because our study did not investigate participants’ reasons for their unwillingness to provide personal information to public health officials, we can only speculate that our participants’ responses were driven by concerns about personal privacy, distrust of governmental experts, misinformation about the goals of contact tracing, or other factors. However, identifying the concerns of rural communities and their residents is critical to addressing them so as to lessen the impact of COVID-19 and provide insight into managing any future pandemics.

To do so, a first step is eliciting what rural residents believe about COVID-19, how it is transmitted, what the risks are of infection, and how they can protect themselves. Research has shown that engaging with rural communities is best done by involving community partners – particularly community health workers and local healthcare providers – rather than outside experts [47–49]. Then messaging could be crafted that addresses the concerns of and is culturally appropriate for specific communities and their members. Involving community members at the ground level will not only ensure their buy-in for the need to collect data but will also promote their willingness to share their data, thereby helping to mitigate public health crises [50].

### Limitations, Strengths, and Future Directions

This study is not without limitations. The primary one is the low response rate. This surprised us given that our earlier mail study about rural residents’ views on governance of their personal health and non-health data had a response rate of 34.1% (accepted). That first survey study with a similar format and questions not discussed herein was completed prior to the emergence of COVID-19. The survey discussed here was mailed in mid-October 2020 when the country was in the midst of a contentious presidential campaign; public health measures were being challenged by politicians at federal and state levels as well as in the media; and vaccines were still under development. In addition to news organizations and pollsters using surveys to track public views and responses to COVID-19, researchers had to resort to surveys due to restrictions caused by the pandemic. With some government agencies reporting lower response rates than usual to surveys and others higher than usual, the evidence is mixed whether people generally were experiencing survey fatigue when they received our survey. Alternatively, some potential respondents may have been turned off by the 6-page document, in which our questions of interest were included or seen the mailing as “junk mail.”

There were other limitations. Our sample is limited to central Pennsylvania. Nearly all participants self-identified as White and non-Hispanic; more than 40% had a high school education or less; fewer than 10% self-identified as liberal; and more than half of respondents were 60+ years of age. While these demographics are representative of rural central Pennsylvania, they are not generalizable to the broader rural population. We have no information on those who declined to participate. We also have no information on why respondents would not share their data or why they were concerned about sharing their data with public health officials investigating a public health threat. Because the survey was not translated to other languages, our sample population is limited to English-speaking individuals. Finally, because we dichotomized our outcome variables, the effects seen may be stronger than had we not dichotomized.

Though our study had limitations, it also had strengths. We examined the perspectives of rural residents on contact tracing and data sharing; the National Center for the Advancement of Translational Science, NIH, has declared rural populations an understudied population, and our data help fill a gap in better understanding the views of this population on an topic relevant to public health and medicine. In addition, our focus on contact tracing and data sharing has thus far not been politicized in the discourse around public health measures important to mitigating the COVID-19 pandemic. Finally, we examined the influence of people’s distrust in public health on their willingness to share data and their concerns about sharing data in the context of contact tracing. We used a validated distrust in healthcare organizations scale as a proxy for distrust in public health, since county and state public health departments can serve as communities’ healthcare organizations.

Our study also points to additional areas for investigation. For instance, it would be helpful for public health officials to understand why individuals who live in rural areas are hesitant to share their data with public health officials investigating public health crises like the COVID-19 pandemic. It would also be important to explore the nature of rural residents’ concerns about sharing those data, so that they could be addressed. Expanding the study population beyond the rural area of central Pennsylvania could result in populations with more ethnic diversity. Finally, the views of individuals living in other regions of Appalachia may differ from those of our populations. Knowing this would enable public health officials to design more customized and therefore more palatable approaches to contact tracing.

## Supplementary material

For supplementary material accompanying this paper visit https://doi.org/10.1017/cts.2023.33. click here to view supplementary material
