# Supplementary material for: Political views and organizational distrust affect rural residents’ willingness to share personal data for COVID-19 contact tracing: A cross-sectional survey study
Source: J Clin Transl Sci. 2023 Mar 27;7(1):e91. doi: 10.1017/cts.2023.33 (PMC10130839; doi:10.1017/cts.2023.33)
Supplement: Supplementary file 1 [file S205986612300033Xsup001.docx]

**Supplemental Methods**

*Scoring of the Distrust in Healthcare Organizations Scale*

The 9 statements in this scale (see Shea et al 2008, Table 3) were on a 5 point Likert scale, Strongly Disagree (1), Disagree (2), Neither Disagree nor Agree (3), Agree (4), and Strongly Agree (5). Responses were summed for questions #2, 4, 5, 8, and 9. Reverse scoring was used for questions #1, 3, 6, and 7. For example, an individual responding Strongly Agree to all questions would have a score of 29.

*Scoring the World Assumption Scale*

The 22 statements in this scale (see Kaler 2009, pages 114-115) were on a 4 point Likert scale, Strongly Disagree (0), Disagree (1), Agree (2), Strongly Agree (3). Responses were summed for questions #1, 3, 9, 10, 12, 18, and 20. Reverse scoring was used for questions #2, 4, 5-8, 11, 13-17, 19, 21, and 22). For example, an individual responding Strongly Agree to all the questions would have a score of 21. (Smith 2016 and personal communication)

*How Variables Were Collapsed*

Age 18-30 + 31-40 = Age <=40

Age 41-50 + 51-60 = Age 41-60

Age 61-70 + 71-80 + >80 = Age >60

Health Status Poor + Fair = Health Status Poor/Fair

Health Status Good = Health Status Good

Health Status Very Good + Excellent = Health Status Very Good/Excellent

Religion Extremely + Very = Religion Extremely/Very

Religion Moderately = Religion Moderately

Religion Slightly + Not at All = Religion Slightly/Not at All

Education Some high school (grades 9-12) + High school graduate or GED = Education High School, GED or Less

Education Some college or technical school = Education Some college or technical school

Education College graduate = Education College graduate

Education Graduate or professional school = Education Graduate or professional school

Employment Retired = Employment Retired

Employment Employed full-time + Employed part-time = Employment Employed

Employment Not current employed + Disabled + Student + Other = Employment Unemployed

Politics Very Conservative + Conservative = Politics Conservative

Politics Moderate = Politics Moderate

Politics Very Liberal + Liberal = Politics Liberal

Politics Other + Prefer not to answer = Politics Other

*Handling of “Prefer not to answer” Responses*

Prefer not to answer was excluded if the N was small and caused issues with analysis as were missing data. All of the exclusions made were less than 5% of the data.

Age Missing were excluded (N=15), Age Prefer not to answer were excluded (N=4)

Gender Missing were excluded (N=16), Gender Prefer not to answer (N=3) and Other (N=1) were excluded

Health Status Missing were excluded (N=22), Health Status Prefer not to answer were excluded (N=5)

Religion Missing were excluded (N=22), Religion Prefer not to answer were excluded (N=13)

Education Missing were excluded (N=29)

Employment Missing were excluded (N=29)

Income Missing were excluded (N=30), Income Prefer not to answer were included (N=174)

Politics Missing were excluded (N=26), Politics Prefer not to answer were included in the Other category (N=175)

Table S1. Frequency and percentages of outcome variables for survey study conducted in fall 2020 of rural patients in central Pennsylvania – not dichotomized

| Question | Frequency (%) |
| --- | --- |
| I would be willing to share my cell phone location data with public health staff investigating COVID-19 cases |  |
| Strongly disagree  Disagree  Neither agree nor disagree  Agree  Strongly agree  Missing | 202 (26.65%)  162 (21.37%)  149 (19.66%)  172 (22.69%)  37 (4.88%)  36 (4.75%) |
| I am concerned that without my permission, my cell phone location data could be shared with public health staff investigating COVID-19 cases. |  |
| Strongly disagree  Disagree  Neither agree nor disagree  Agree  Strongly agree  Missing | 37 (4.88%)  132 (17.41%)  170 (22.43%)  258 (34.04%)  125 (16.49%)  36 (4.75%) |
| I would be willing to share data (names, addresses, phone numbers) of people with whom I have had recent in-person contact with public health staff investigating COVID-19 cases. |  |
| Strongly disagree  Disagree  Neither agree nor disagree  Agree  Strongly agree  Missing | 106 (13.98%)  146 (19.26%)  142 (18.73%)  284 (37.47%)  49 (6.46%)  31 (4.09%) |
| I am concerned that the personal data I share with public health staff may be used for purposes other than investigating COVID-19 cases. |  |
| Strongly disagree  Disagree  Neither agree nor disagree  Agree  Strongly agree  Missing | 43 (5.67%)  130 (17.15%)  164 (21.64%)  268 (35.36%)  126 (16.62%)  27 (3.56%) |

Table S2: Characteristics of respondents in relation to contact tracing and data sharing as Odds Ratios (95% Confidence Intervals), Bivariate analysis for survey study conducted in fall 2020 of rural patients in central Pennsylvania

| Characteristic | I would be willing to share my cell phone location data with public health staff investigating COVID-19 cases. | I am concerned that without my permission, my cell phone location data could be shared with public health staff investigating COVID-10 cases. | I would be willing to share data (names, addresses, phone #s) of people with whom I have had recent in-person contact with public health staff investigating COIVID-10 cases. | I am concerned that the personal data I share with public health staff may be used for purposes other than investigating COVID-19. |
| --- | --- | --- | --- | --- |
|  |  |  |  |  |
| Gender |  |  |  |  |
| Male | 1 | 1 | 1 | 1 |
| Female | 0.75 (0.54-1.04), p=0.174 | 0.83 (0.62-1.12), p=0.295 | 0.87 (0.65-1.17), p=0.360 | 0.68 (0.50-0.92), **p=0.046** |
|  |  |  |  |  |
| Health |  |  |  |  |
| Poor/Fair | 1 | 1 | 1 | 1 |
| Good | 1.45 (0.96-2.17), p=0.243 | 0.79 (0.55-1.15), p=0.243 | 1.31 (0.90-1.90), p=0.243 | 0.80 (0.55-1.16), p=0.243 |
| Very good/Excellent | 0.83 (0.53-1.30), p=0.636 | 0.91 (0.61-1.37), p=0.636 | 1.13 (0.76-1.67), p=0.636 | 0.78 (0.53-1.15), p=0.636 |
|  |  |  |  |  |
| Age |  |  |  |  |
| <40 | 1 | 1 | 1 | 1 |
| 41-60 | 1.08 (0.60-1.95), p=0.984 | 1.33 (0.81-2.19), p=0.984 | 1.01 (0.60-1.69), p=0.984 | 1.18 (0.72-1.94), p=0.984 |
| >60 | 1.71 (1.01-2.89), p=0.093 | 1.04 (0.66-1.63), p=0.862 | 2.08 (1.31-3.31), **p=0.008** | 1.19 (0.76-1.87), p=0.593 |
|  |  |  |  |  |
| Importance of religion, spirituality |  |  |  |  |
| Extremely/Very | 0.52 (0.34-0.80), **p=0.012** | 1.60 (1.05-2.43), **p=0.037** | 0.82 (0.55-1.24), p=0.351 | 1.60 (1.06-2.41), **p=0.037** |
| Moderately | 0.69 (0.42-1.15), p=0.286 | 1.92 (1.17-3.14), **p=0.038** | 0.73 (0.45-1.20), p=0.286 | 1.29 (0.80-2.09), p=0.302 |
| Slightly/Not at all | 1 | 1 | 1 | 1 |
|  |  |  |  |  |
| Education |  |  |  |  |
| High school/GED or less | 0.45 (0.27-0.73), **p=0.003** | 0.99 (0.63-1.56), p=0.968 | 0.40 (0.25-0.63), **p<0.001** | 1.48 (0.94-2.31), p=0.118 |
| Technical school or some college | 0.86 (0.52-1.43), p=0.569 | 0.86 (0.53-1.41), p=0.569 | 0.60 (0.37-0.98), p=0.172 | 1.27 (0.78-2.06), p=0.569 |
| College graduate | 0.98 (0.58-1.66), p=0.941 | 0.84 (0.50-1.39), p=0.680 | 0.61 (0.36-1.01), p=0.214 | 1.18 (0.72-1.96), p=0.680 |
| Graduate or professional school | 1 | 1 | 1 | 1 |
|  |  |  |  |  |
| Employment status |  |  |  |  |
| Not employed | 1 | 1 | 1 | 1 |
| Employed | 0.87 (0.53-1.45), p=0.599 | 1.25 (0.80-1.96), p=0.599 | 1.49 (0.93-2.37), p=0.377 | 0.88 (0.57-1.37), p=0.599 |
| Retired | 1.28 (0.79-2.07), p=0.622 | 1.08 (0.70-1.66), p=0.972 | 2.27 (1.45-3.56), **p= 0.001** | 1.01 (0.66-1.55), p=0.972 |
|  |  |  |  |  |
| Total household income |  |  |  |  |
| $30,000 or less | 1.02 (0.55-1.86), p=0.960 | 1.26 (0.72-2.20), p=0.622 | 0.81 (0.47-1.41), p=0.622 | 1.23 (0.71-2.13), p=0.622 |
| $31,000 - $50,000 | 1.21 (0.68-2.18), p=0.790 | 0.65 (0.38-1.12), p=0.493 | 0.97 (0.56-1.67), p=0.905 | 1.16 (0.67-2.00), p=0.790 |
| $51,000 - $70,000 | 1.00 (0.55-1.82), p=0.990 | 1.07 (0.62-1.86), p=0.990 | 0.92 (0.53-1.59), p=0.990 | 1.54 (0.89-2.66), p=0.492 |
| $71,000 - $100,000 | 1.58 (0.86-2.91), p=0.563 | 0.86 (0.48-1.53), p=0.880 | 0.96 (0.54-1.71), p=0.880 | 0.90 (0.51-1.61), p=0.880 |
| Greater than $100,000 | 1 | 1 | 1 | 1 |
| Prefer not to answer | 0.57 (0.32-1.04), p=0.137 | 1.15 (0.69-1.94), p=0.589 | 0.50 (0.30-0.85), **p=0.040** | 1.53 (0.91-2.56), p=0.141 |
|  |  |  |  |  |
| Political values |  |  |  |  |
| Very conservative, Conservative | 0.20 (0.11-0.37**), p<0.001** | 4.70 (2.43-9.10), **p<0.001** | 0.20 (0.10-0.38), **p<0.001** | 3.07 (1.67-5.61), **p<0.001** |
| Moderate | 0.44 (0.24-0.82), **p=0.030** | 2.37 (1.18-4.74), **p=0.030** | 0.50 (0.25-1.00), p=0.066 | 1.57 (0.83-2.98), p=0.166 |
| Liberal, Very liberal | 1 | 1 | 1 | 1 |
| Prefer not to answer | 0.15 (0.08-0.29), **p<0.001** | 5.66 (2.84-11.30), **p<0.001** | 0.15 (0.08-0.30), **p<0.001** | 3.33 (1.77-6.28), **p<0.001** |
|  |  |  |  |  |
| DHO Score | 0.75 (0.64-0.89), **p=0.002** | 1.21 (1.05-1.40), **p=0.013** | 0.86 (0.75-0.99), **p=0.035** | 1.26 (1.09-1.45), p**=0.003** |
|  |  |  |  |  |
| WAQ | 1.17 (1.02-1.35), **p=0.039** | 0.85 (0.74-0.97), **p=0.039** | 1.16 (1.02-1.32), **p=0.039** | 0.87 (0.77-0.99), **p=0.039** |
|  |  |  |  |  |
| * Odds ratios, 95% confidence limits, and p-values from binomial logistic regression; 1 = Strongly Disagree/ Disagree/ Neither; 0 = Agree/Strongly Agree | | | | |
